# Malaria and obesity: obese mice are resistant to cerebral malaria

**DOI:** 10.1186/1475-2875-7-81

**Published:** 2008-05-19

**Authors:** Vincent Robert, Catherine Bourgouin, Delphine Depoix, Catherine Thouvenot, Marie-Noëlle Lombard, Philippe Grellier

**Affiliations:** 1 Institut de Recherche pour le Développement, Unité de Recherche 77 and Unité de Recherche 16, 213 rue La Fayette, 75480 Paris cedex 10, France; 2 Unité Scientifique du Muséum 504, Muséum National d'Histoire Naturelle, 61 rue Buffon, 75231 Paris cedex 05, France; 3 Centre de Production et d'Infection des *Anopheles*, Institut Pasteur, 28 rue du Dr Roux, 75724 Paris cedex 15, France

## Abstract

**Background:**

The relationship between malaria and obesity are largely unknown. This is partly due to the fact that malaria occurs mainly in tropical areas where, until recently, obesity was not prevalent. It now appears, however, that obesity is emerging as a problem in developing countries. To investigate the possible role of obesity on the host-parasite response to malarial infection, this study applied a murine model, which uses the existence of genetically well characterized obese mice.

**Methods:**

The receptivity of obese homozygous *ob/ob *mice was compared to the receptivity of control heterozygous *ob*/+ lean mice after a single injection of *Plasmodium berghei *ANKA sporozoites. Both parasitaemia and mortality in response to infection were recorded.

**Results:**

The control mice developed the expected rapid neurological syndromes associated with the ANKA strain, leading to death after six days, in absence of high parasitaemia. The obese mice, on the other hand, did not develop cerebral malaria and responded with increasing parasitaemia, which produced severe anemia leading to death 18–25 days after injection.

**Conclusion:**

The observed major differences in outward symptoms for malarial infection in obese versus control mice indicate a link between obesity and resistance to the infection which could be addressed by malariologists studying human malaria.

## Introduction

Obesity, mainly characterized by an excess of adipose tissue, is associated with high risks of developing several pathologies that have disastrous consequences. This health condition is of paramount importance for public health in developed countries. For example, in the USA more than half of the population is considered to be obese according to the WHO criteria. Obesity has also been identified as an emerging condition in developing countries, especially in towns of tropical areas [1,2].

Crude NCBI-PubMed searches with "obesity" and "malaria" yielded 107,545 and 46,653 references, respectively. However, association of the two terms produced only 17 entries, two of which partially addressed the issue [3,4], indicating that the two communities of researchers occupy distinct scientific niches that do not overlap. A limited survey amongst fellow malariologists also revealed the lack of bridges between the two disciplines. Finally, a search in the French database of all recorded malaria cases (about 45,000) was eloquent: the bodyweight of the studied patients was simply not recorded.

Several studies have been performed on malaria and malnutrition in humans [5-7]. They strongly suggest that malaria is the major contributor to growth retardation in children living in endemic areas. Evidence from observational cohorts indicated that malnutrition decreases the susceptibility to malaria, and that this may be due to an interaction between the parasite and the host immune system [8]. At the same time, it can also worsen the prognosis of malaria [9]. However, the point at which malnutrition ceases to be protective and becomes an adverse prognosticator is not clear [10]. Experiments with mice demonstrate that nutritional status interferes with the expression of malarial disease. Lean mice fed on a normal diet (25% protein) suffered severe parasitaemia and died within two weeks when infected with a lethal strain of *Plasmodium yoelii*, whereas mice fed on low-protein diet survived without apparent parasitaemia. This response has been related to an enhanced innate immunity in mice fed on low-protein diet [11]. In another study, *P. berghei *malaria sporozoites were less infective in knock out LDLR-/- mice (low density lipoprotein receptors) maintained on a high fat diet, as compared to littermates maintained on a normal diet [12]. While some studies suggest a link between nutritional status (malnutrition) and the evolution of malaria infections, little is known about the relationship of obesity vis-à-vis the prevalence or evolution of malarial infections in human [13]. Considering the increasing world concern about obesity, on the one hand, and malaria on the other, any relationship between both clinical statuses would be of great significance.

This study investigated the possibility of such a relationship, using the murine model *ob/ob*, which provides valuable tools for studying obesity. The obese *ob/ob *mice (C57BL/6 background) have developed a spontaneous recessive and nonsense punctual mutation in the *leptin *gene which, in the mutant *ob/ob *homozygote, encodes for a truncated non-functional protein, resulting in the absence of retro-control on hunger. These leptin-deficient mice exhibit irrepressive feeding behaviour and develop a patent obesity [14]. Leptin, a hormone mainly produced by adipocytes, is a central mediator of the neuroendocrine pathways involved in the control of food intake, basal metabolism and reproductive function. As a consequence, *ob/ob *mice exhibit a hyperlipidemia caused by an increase in hepatic triglyceride synthesis and secretion [15]. The obese mice also develop hyperglycaemia and type 2 diabetes, which occurs as a consequence of obesity rather than a primary effect of leptin. Recent evidence also indicates that leptin acts as a proinflammatory cytokine [16].

A literature search indicates that obese mice have not been experimentally infected by malaria parasites previously. Obesity could play a protective, neutral or permissive role in the evolution of malarial infections and subsequent patho-physiological manifestations. However, many factors can interfere. Here, using the ANKA strain of *P. berghei *which induces cerebral malaria in C57BL/6 mice, it has been shown that the outcome of malaria infection differs dramatically between obese and control lean mice. Contrary to the control mice, the obese mice did not develop cerebral malaria.

## Materials and methods

Nine eleven week-old C57BL/6 homozygous *ob/ob *male mice were obtained from a rearing centre (R. Janvier, 53940 Le Genest-Saint-Isle, France). The controls were heterozygote *ob*/+ mice from the same rearing centre and of the same sex and age. The mice were infected by intravenous inoculation of either 50,000 or 100,000 *P. berghei *(clone 15cy1 of the ANKA strain) sporozoites isolated from laboratory infected *Anopheles stephensi*. The mean weights of the obese and control mice at day 0 (the day of inoculation) were 47.1 g and 25.4 g, respectively. Parasitaemia was monitored daily by microscopic examination of Giemsa stained-thin blood smears. Parasite counts were performed at 1,000× magnification on smear regions with an estimation of 1,000 erythrocytes per field, on 20–100 microscopic fields for parasitaemias ≤ 3%. For higher parasitaemias, usually associated with anaemia, examinations were performed on 4–10 fields with parallel counts of parasitized and unparasitized erythrocytes. In the absence of observed parasites, thin smears were examined over 30 minutes. Parasite counts were expressed as percentage of erythrocytes. Two independent experimental series have been performed; the results are in perfect agreement and are, therefore, presented as a single set, except in Table 1.

**Table 1:** Parasitaemias of *Plasmodium berghei *ANKA-infected C57BL/6 mice, as % of parasitized red blood cell.

	Number of sporozoites per mouse	Day of infection	2	3	4	5	6	7	8	10	14	19
Control	50,000	Number of mice	9	9	9	9	9	2	2	2	2	2
*ob*/+		Mean parasitaemia	0.000	0.011	0.221	1.61	3.01	2.83	3.42	7.37	32.4	39.9
		Standard deviation	--	0.006	0.109	0.29	1.07	1.38	0.44	3.09	21.2	7.8
Obese	50,000	Number of mice	5	5	5	5	5	5	5	5	5	5
*ob/ob*		Mean parasitaemia	0.000	0.014	0.110	1.57	3.34	3.83	4.81	4.83	11.2	18.3
		Standard deviation	--	0.009	0.113	0.40	0.40	0.94	1.35	1.16	6.07	5.78
Obese	100,000	Number of mice	4	4	4	4	4	4	4	4	4	4
*ob/ob*		Mean parasitaemia	0.000	0.027	0.321	1.96	3.94	4.61	3.81	4.16	10.8	23.9
		Standard deviation	--	0.025	0.155	0.51	0.96	0.85	1.35	0.28	6.12	5.11

Results of the first experimental series of infection, comparing the doses of injected sporozoites.

## Results

**Figure 1:**
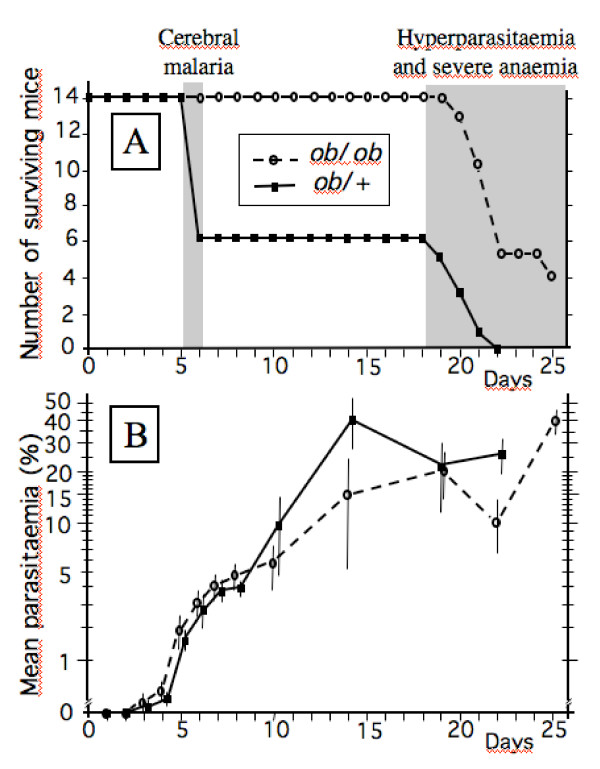
**A- Survival curves for obese *ob/ob *mice and control *ob*/+ mice after infection by *Plasmodium berghei *ANKA at day 0; B- Parasitaemias with standard deviations, as % of parasitized red blood cell.** (Note the Log scale for vertical axis.) Continuous line, *ob*/+; broken line, *ob/ob*.

All injected mice (14 obese *ob/ob *and 14 control *ob/*+) developed infection with parasites observed in the blood as early as day 3 post infection (PI). Among the control mice, 8 out of 14 died from cerebral malaria at day 6 PI without precursory clinical signs (Fig. 1-A) and with parasite blood densities of around 3%. Within this control group, the mice had similar parasitaemias at any day whatever the outcome of their symptoms; at day 6, mean parasitaemia was 2.73% in mice that survived (6/14) and 3.18% in mice that died a few hours later (8/14), P = 0.44 by the Man-Whitney *U *test. The surviving mice ultimately died after day 19 from severe anaemia resulting from high parasite densities reaching up to 40% (Fig. 1-B). At day 18 PI, their mean body weight was 20.2 g.

In contrast, the obese mice did not present any clinical sign of cerebral malaria (behavioral changes or coma). They died from severe anaemia between days 18 and 25 PI (those surviving at day 25 were in the final phase and sacrificed). Cox proportional hazard indicated a P-value of 2.8 × 10^-5 ^and a death risk factor, at any moment, 7.34 times lower for obese mice. The parasitaemia was higher in the obese mice than in the control mice from day 3 to day 8 PI (Fig. 1-B), but the daily differences were never significant (0.98 > P > 0.07 by the Mann-Whitney *U *test), except at day 3 (0.017 parasitized erythrocytes per 100 erythrocytes in the control mice vs. 0.039 in the obese mice; P = 0.032). At day 18 PI, the mean body weight of the surviving *ob/ob *mice was 45.8 g.

The amount of injected sporozoites per mouse (50,000 or 100,000) had only transitory consequences on parasitaemias that were detectable only at days 3 and 4 PI (Table 1), indicating that doubling the sporozoite dose proportionally to the obese weight does not affect the observed responses in terms of mortality.

## Discussion

This study appears to provide the first indication that leptin-deficient *ob/ob *obese mice are permissive for the infection by *Plasmodium berghei *ANKA and resistant to cerebral malaria. This resistance cannot be explained solely by the larger size of these mice, as weight-adjusted doses of sporozoites were given. The protection of the obese mice with respect to cerebral malaria was unexpected, since they share the same C57BL/6 genetic background as the lean mice which are well known to be susceptible to cerebral malaria, usually dying from typical neurological symptoms between days 6–9 after infection [17]. The susceptibility to cerebral malaria of this strain is confirmed by observations with the control lean heterozygote *ob*/+ mice.

Cerebral malaria induced by *P. berghei *ANKA is known to be under genetic regulation, but the mechanisms leading to protection versus death from cerebral malaria are not yet fully understood in normal non-obese mice [18]. Using mice of different genetic backgrounds which are either cerebral malaria (CM) sensitive or CM resistant, and comparing gene expression profiling, Delahaye *et al *[19,20] identified a large set of genes that discriminates between CM sensitive and resistant mice, including genes involved in regulating immune responses. Not surprisingly, the obese phenotype affects the expression of a large panel of genes, mainly involved in metabolic pathways possibly inducing resistance to CM [21,22]. The *leptin *gene mutation in *ob/ob *is associated with observed protection against CM but, considering the complex and pleiotrophic effect of the leptin mutation in *ob/ob *mice, it is hard to speculate whether the unique mutation of the *leptin *gene would be totally responsible, per se, for the observed CM resistance phenotype in obese mice.

Several lines of evidences can be related to possible mechanisms involved in this protection. Among them are many related to the nutritional status and plasmatic lipid composition involved in immune responses. So the deep modifications of the lipid metabolism in *ob/ob *mice can have implications. Obese mice exhibit a hyperlipidemia caused by an increase in hepatic triglyceride synthesis and secretion [15]. Interestingly, it has been demonstrated that injection of fatty acids during the first three days after infection protected C57BL/6 lean mice infected by *P. berghei *ANKA from cerebral symptoms [23]. Regulation of glycemia is also affected in obese mice, resulting in hyperglycemia that may compensate the hypoglycemia commonly observed in severe or complicated malaria attacks. Elased *et al *[24] observed that infection with *P. yoelii *induces hypoglycemia in normal mice and normalizes the hyperglycemia in moderately diabetic mice. In contrast, blood glucose concentrations significantly rose in mice exhibiting neurological symptoms during infection with *P. berghei *ANKA [25].

On the other hand, *ob/ob *mice are leptin-deficient, and this could be one of the key factors involved in the observed resistance. Indeed, a progressive increase of leptin concentration was observed in *P. berghei *(Swiss Tropical Institute strain)-infected C57BL/6 lean mice, and the level of leptin increased five times when compared with non-infected control mice, six days after infection [26]. Leptin is involved in protective mechanisms, allowing an organism to deal with the potentially auto-aggressive effects of its immune system [27]. In other experiments, injections of leptin were claimed to protect against TNF toxicity in *ob/ob *but not in +/+ mice [28]; therefore, it is possible that leptin protects against cerebral malaria by damping down TNF-induced pathology. Similar in structure to interleukin 2, an important T-cell growth factor, leptin modifies proinflammatory immune responses and may provide a key link between nutritional deficiency and immune dysfunction. Obese *ob/ob *mice have impaired cell-mediated immunity and a propensity to develop Th2 rather than Th1 immune responses [29]. A decrease in the Th1: Th2 ratio could be related to the observed resistance in obese mice because cerebral malaria due to *P. berghei *ANKA involves the Th1 cytokines TNF-α and INF-γ in the regulatory cascade controlling inflammatory responses after malarial infections [30].

Leptin can also regulate aspects of hemopoiesis, inducing proliferation, differentiation and functional activation of hemopoietic cells, but it has a weak impact on the bone-marrow nucleated erythroid cells [31]. In other words, the severe anaemia observed in infected obese mice is a direct consequence of erythrocyte destruction by parasite proliferation, rather than leptin deficiency. Similar anaemia was observed in the few control mice that did not develop cerebral malaria.

While this study addressed the outcome of malaria in *ob/ob *mice, which are non-diabetic until the fourth week [32], it questions what would be the response of *db/db *diabetic mice, which also present obese phenotype. Indeed, drug-induced diabetic mice were shown to better control parasitaemia with enhanced phagocytic activity compared to normal mice [33]. Apparent normalization of blood glucose concentration was also observed in a diabetic patient suffering from *P. falciparum *malaria [34]. On the contrary, in mice exhibiting neurological symptoms during infection with *P. berghei *ANKA, blood glucose concentrations were significantly raised in line with TNF levels [25].

Beyond the malaria symptoms previously discussed, parasite density is worth considering. The data from this study showing higher parasitaemia in *ob/ob *mice during days 3–8 after infection are in agreement with what has been observed in obese *fa/fa *Zucker rats infected by *P. yoelii yoelli *presenting higher parasitaemias than heterozygous control rats. The higher and significant parasitaemia in *ob/ob *mice at day 3 questions a possible increased productivity of hepatic merozoites, as observed in single gene mutated Zucker rats [35].

It is noteworthy that mutations affecting *leptin *exist in humans, but with inconstant phenotypic manifestations in terms of obesity [36]. Although experimental cerebral malaria models cannot reproduce all the features of human cerebral malaria, several observations in mice have been extended and confirmed in the human disease [37]. They include immunological responses, blood-barrier function, histopathological features and expression of molecules in the brain and the retina, biochemical changes in the brain and behavioral changes. Events resulting in the development of human cerebral malaria complications are clearly multi-factorial, encompassing a dynamic interaction between sequestration, inflammation and homeostasis, in a complex syndrome leading to microcirculatory dysfunction [30,38].

## Conclusion

Control heterozygous mice infected with *P. bergei *ANKA developed rapid neurological syndromes leading to death, without high parasitaemia. By contrast, obese *ob/ob *mice did not develop cerebral malaria and presented an increasing parasitaemia resulting in severe anaemia. Although it is not possible to make inferences from mice to humans from these observations, the study highlights the need to recognize and address a possible link between obesity and its effect on the response to malarial infection in human populations.

## Authors' contributions

VR, CB, M–NL and PG conceived the study. CT infected mosquitoes and provided the sporozoites. VR and DD infected the mice and followed-up the infected mice. VR carried out the microscopic studies. All authors wrote the paper and gave final approval of the version to be published.
